# Screening of Fish Cell Lines for Piscine Orthoreovirus-1 (PRV-1) Amplification: Identification of the Non-Supportive PRV-1 Invitrome

**DOI:** 10.3390/pathogens9100833

**Published:** 2020-10-12

**Authors:** Phuc H. Pham, Ehab Misk, Fotini Papazotos, Ginny Jones, Mark P. Polinski, Elena Contador, Spencer Russell, Kyle A. Garver, John S. Lumsden, Niels C. Bols

**Affiliations:** 1 Department of Biology, University of Waterloo, Waterloo, ON N2L 3G1, Canada; fpapazot@uwaterloo.ca (F.P.); ncbols@uwaterloo.ca (N.C.B.); 2 Department of Pathobiology, Ontario Veterinary College, University of Guelph, Guelph, ON N1G 2W1, Canada; elenacontador@icloud.com (E.C.); jsl@uoguelph.ca (J.S.L.); 3 Huntsman Marine Science Centre, St. Andrews, NB E5B 2L7, Canada; ehab.misk@huntsmanmarine.ca; 4 Elanco Canada Limited, Aqua Vaccines R&D, Charlottetown, PE C1E 2A7, Canada; jones_ginny@elanco.com; 5 Pacific Biological Station, Department of Fisheries and Oceans, Nanaimo, BC V9T 6N7, Canada; Mark.Polinski@dfo-mpo.gc.ca (M.P.P.); Kyle.Garver@dfo-mpo.gc.ca (K.A.G.); 6 Fisheries and Aquaculture, Vancouver Island University, Nanaimo, BC V9R 5S5, Canada; spencer.russell@viu.ca

**Keywords:** piscine reovirus, PRV-1, fish cell lines, non-supportive, heart and skeletal muscle inflammation, HSMI, invitrome, RNA virus, Atlantic salmon

## Abstract

Piscine reovirus (PRV) is the causative agent of heart and skeletal muscle inflammation (HSMI), which is detrimental to Atlantic Salmon (AS) aquaculture, but so far has not been cultivatable, which impedes studying the disease and developing a vaccine. Homogenates of head kidney and red blood cells (RBC) from AS in which PRV-1 had been detected were applied to fish cell lines. The cell lines were from embryos, and from brain, blood, fin, gill, gonads, gut, heart, kidney, liver, skin, and spleen, and had the shapes of endothelial, epithelial, fibroblast, and macrophage cells. Most cell lines were derived from the Neopterygii subclass of fish, but one was from subclass Chondrostei. Cultures were examined by phase contrast microscopy for appearance, and by quantitative polymerase chain reaction (qPCR) for PRV-1 RNA amplification and for the capacity to transfer any changes to new cultures. No changes in appearance and Ct values were observed consistently or transferable to new cultures. Therefore, 31 cell lines examined were unable to support PRV-1 amplification and are described as belonging to the non-supportive PRV-1 invitrome. However, these investigations and cell lines can contribute to understanding PRV-1 cellular and host tropism, and the interactions between virus-infected and bystander cells.

## 1. Introduction

**Figure 1:**
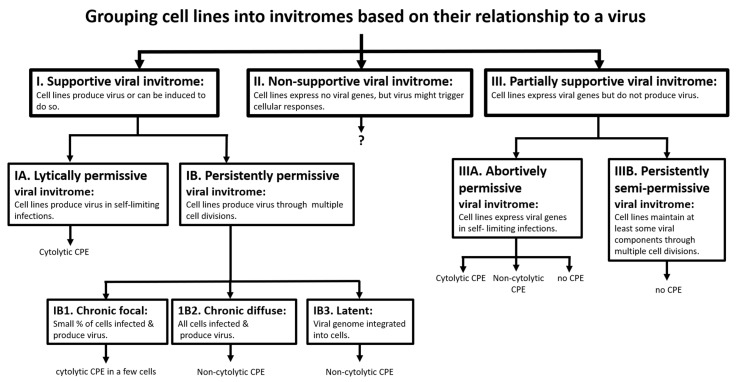
An informal classification tree for dividing all animal cell lines into invitromes based on how they interact with a specific virus. The diagram presents the range of potential interactions between a virus and cell lines, but for a particular virus, only some of these interactions might take place. Cell lines that produce virions are members of the supportive viral invitrome (left rectangle labeled with Roman numeral I), whereas cell lines that neither produce virions nor express viral genes belong to the non-supportive viral invitrome (center rectangle labeled with Roman numeral II). Cell lines that express viral genes but do not produce virions are in the partially supportive invitrome (right rectangle labeled with Roman numeral III). The supportive viral invitrome (I) can be subdivided into the lytically permissive invitrome (IA), in which cell lines produce virions in self-limiting infections, and the persistently permissive viral invitrome (IB), in which cell lines produce virions through multiple cell divisions or can be induced after multiple cell divisions to produce virions. Subdivisions of IB are chronic focal (IB1), chronic diffuse (IB2), and latent (IB3) invitromes, and are defined in the diagram. Finally, in the partially supportive viral invitrome (III), cells support the expression of viral genes but not the full viral life cycle and virion production. This group can be divided into the abortively permissive viral invitrome (IIIA), in which the cell lines become infected but the infection is aborted before virions are produced, and the persistently semi-permissive invitrome (IIIB) in which the cell lines continuously maintain viral component(s) but neither produce nor can be induced to produce virions.

The purpose of this work was to screen fish cell lines for their ability to support the life cycle of, and amplify, piscine reovirus (PRV), in the hope that at least one would be found and then could be used to study heart and skeletal muscle inflammation (HSMI). PRV is the primary causative agent of HSMI in farmed Atlantic salmon (AS) [1,2,3]. To date, no cell lines have been reported to support PRV amplification. For broadly studying viral diseases, cell lines susceptible to the etiological agent and supportive of its replication are invaluable, being used for multiple purposes from diagnostics to vaccines [4]. Yet, in some cases, efficient cell culture systems have been identified only after decades of research [5]. Searching for cell lines suitable for studying viral diseases is a general problem, but common features of these searches often appear hidden by the uniqueness of each pathogen. Therefore, a broad outline for viewing the diversity of fish cell lines (Table 1) and of fish virus/interactions (Figure 1) are briefly reviewed below and is the context for discussing the results of the screen for PRV supportive cell lines. 

Screening cell lines for their ability to produce a new virus is challenging in part because of the surprisingly large number of animal cell lines. The online data base, Cellosaurus, has over 100,000 cell lines, which includes over 500 from fish [6]. To make using cell lines easier to think about and apply to research problems, the term “invitrome” has been proposed to describe a grouping of cell lines around a particular theme [7]. The animal invitrome constitutes all cell lines from metazoans that have been reported in scientific communications. These can be subdivided based on either the properties of the species and organ from which the cell line was started, or on the characteristics of the cell line after its establishment. This is illustrated in Table 1, which lists the cell lines of this study and divides them into invitromes based on the properties of the fish (columns I.A1 to 3) and starting tissues (column I.B1) from which they were derived.

To date, the ability to support PRV replication has been investigated in cell lines from AS (ASK, SHK-1), rainbow trout (RTG-2), Chinook salmon (CHSE-214), common carp (CCB), fathead minnow (EPC, FHM) and orange spotted grouper (GF-1) [8,9,10,11]. Based on the life history and natural distribution of these species, these eight cell lines belong to anadromous (ASK, SHK-1, RTG-2, CHSE-214), freshwater (CCB, EPC, FHM), and marine invitromes (GF-1), and to North Atlantic (ASH, SHK-1), North Pacific Ocean (RTG-2, CHSE-214), Indo-Pacific Ocean (GF-1), Great Lakes Basin (EPC, FHM), and Asia-Europe (CCB) invitromes. Their anatomical origins were kidney (ASK, SHK-1), gonad (RTG-2), skin (EPC, FHM), brain (CCB), fin (GF-1), and embryo (CHSE-214).

Another way that the fish invitrome can be subdivided is on the basis of the properties of the cell lines after they have been established (heading II in Table 1). One property is cell line availability. For example, the curated invitrome constitutes all the cell lines cataloged in repositories, such as the American Type Culture Collection (ATCC) [7]. Most of these would be available commercially, although some are owned by private companies and might require a more comprehensive legal agreement. All the cell lines that have been used to investigate PRV have been from the curated invitrome. The curated fish invitrome is relatively small compared to the informally shared invitrome, which is all the cell lines described in the literature but are not in repositories and instead are informally shared between researchers [7]. These have yet to be used to study PRV and HSMI homogenates.

Vulnerability to a particular virus can be expected to vary between the cell lines of a cell line collection (invitrome) (Figure 1). The life cycle of a virus can either be fully, partially, or not supported by a cell line. Application of text book definitions of susceptibility and resistance to cell lines works well when functional receptors for a virus are reasonably understood [12,13,14], but is awkward when a broad taxonomical range is being screened, and knowledge of receptors for the virus in most species is inadequate. Therefore, cell lines will be described as belonging to either supportive, partially supportive, or non-supportive invitromes (Figure 1). These are expanded on below, using piscine cells and viruses as examples.

Cell lines producing infectious virions belong to the supportive invitrome for a particular virus and can be described as being either lytically or persistently permissive (Figure 1). Lytically permissive cell lines produce virus in self-limiting infections: the viral life cycle is completed, virions are released, and cells die. The death or cytolysis of cells is an easily observable CPE endpoint, making these cell lines useful diagnostic tools, such as CHSE-214 for IPNV [15]. Cytolysis also limits the life of infected cultures, but close scrutiny of some cultures can reveal a few surviving cells. These presumably have the features to become infected but also the capacity to recover, making them very interesting but difficult to study cells [16]. Persistently permissive cell lines produce virus through multiple cell divisions or passages. Persistent cell cultures can be of three types: latent, chronic focal, and chronic diffuse [17]. For these, changes in culture appearance or cytopathic effect (CPE) does not take place except for a small amount in chronic focal cultures where a few cells undergo cytolysis and release virions. Examples of latent cultures have been identified with several fish cell lines and C-type retrovirus [18]. Chronic diffuse cultures have been noted with CHSE-214 and at least two RNA viruses, IPNV [19], and cutthroat trout virus (CTV) [20]. Chronic focal cell lines, which have a very low percentage of cells producing virus and have been referred to as carrier cultures [21], have been prepared from virally infected fish, including barramundi with a betanodavirus [22] and snakehead with a birnavirus [23].

The partially supportive invitrome is made up of cell lines that become infected but fail to produce infectious virions and has two branches (Figure 1). One branch consists of cell lines expressing viral gene(s) in self-limiting infections and so is termed abortively permissive. An example is the rainbow trout monocyte/macrophage cell line, RTS11, with two Ranaviruses: grouper iridovirus (GIV) and frog virus (FV-3). RTS11 express early viral transcripts for GIV and FV-3 but rapidly undergo cytolysis before any virus is produced [24,25]. Whether infections in the abortively permissive invitrome could be limited without the expression of cell death or be accompanied by another form of CPE is currently unknown but are possibilities (Figure 1). The second branch consists of cell lines continuously maintaining viral components while proliferating but never producing infectious virions, and so are termed persistently semi-permissive. An example is the marbled eel cell line, DMEPF-5, and adomavirus. These cells retain adomavirus nucleic acid but do not appear to produce the virus [26].

Finally, some cell lines show no evidence of viral replication or gene expression and belong to the non-supportive viral invitrome (Figure 1). These cells still might sense virions and make responses to them, but the virions are passive agents. For example, although not infecting vertebrate cells [27], some bacteriophages seem to elicit responses in mammalian cells [28,29]. Non- supportive cell lines are used occasionally to study cellular and host tropisms, but an additional important use is emerging. During a viral infection, an animal will have both infected cells and non-infected cells (bystanders). How infected and bystander cells interact is only beginning to be explored [30,31]. Non-supportive cell lines should be a valuable source of bystanders for in vitro studies on the interactions between infected and non-infected cells. 

To date, attempts to cultivate PRV from fish homogenates have been equivocal. The first tries used ASK, CHSE-214, FHM, EPC and GF-1 with homogenates of heart and kidney from Norwegian farmed AS that had been diagnosed with HSMI [9,10,11]. Subsequently, kidney and spleen homogenates from Canadian farmed AS that had tested positive for PRV RNA were applied to CHSE-214, EPC and GF-1 [32]. Additionally, ASK, CCB, CHSE-214, GF-1, and RTG-2 were exposed to brain, kidney, liver and spleen homogenates from jaundiced Chinook salmon that were positive for PRV [8]. The most promising results were seen in GF-1 cultures, with CPE manifesting as vacuoles, and with the culture supernatant transmitting the CPE to new cultures and causing HSMI symptoms in fish [10]. However, other studies with GF-1 found that PRV RNA copy number as determined by quantitative polymerase chain reaction (qPCR) did not increase over 6 days despite the appearance of CPE, and the CPE disappeared upon passaging [8,32]. CPE has been reported with other cell lines. Vacuoles were seen with ASK [11], and small foci of rounded cells with some detachment were observed in cultures of CCB, EPC and FHM [8,9]. However, three studies report no CPE with CHSE-214 [8,11,32]. The conclusion is that PRV is uncultivatable.

Therefore, the search for a cell culture system to support the PRV-1 life cycle has been expanded. Homogenates of primarily head kidney and RBC from PRV-1-infected AS were applied to cell lines from 14 fish species and 11 organs, as well as from embryos. Additionally, a cell line was derived from a PRV-1 infected Atlantic salmon. PRV-1 amplification was evaluated by monitoring cultures for increasing viral RNA quantity (Ct) and/or changes in cellular appearance over time. From these, the 31 cell lines were concluded to be non-supportive to PRV-1. With a few of these, the potential value of non-supportive cell lines for studying infected cell/bystander cell interactions in HSMI was demonstrated by measuring, through Western blotting, the induction of myxovirus-resistant protein (Mx), a classic antiviral effector [33], by RBC homogenates.

## 2. Results

### 2.1. Screening 30 Cell Lines with PRV-1 Containing Homogenates for Capacity to Support PRV-1 Amplification In Vitro 

This section provides an overview of the screening of 30 cell lines for capacity to support PRV-1 amplification in vitro. The detailed screening results will be described in individual subsections below. Three isolates of PRV-1 [Chilean (PRV-1-unknown subtype), Canadian (PRV-1a), and Norwegian (PRV-1b)] were screened for capacity to replicate and amplify in 30 fish cell lines (Table 1; one cell line from Table 1 was not screened with PRV-1 containing homogenate in vitro). Most cell lines were previously established (either curated or informally shared), while six were newly developed; the status of each cell line is indicated in Table 1. The screening was performed over four years (from 2014 to 2018) as the PRV-1 source materials [head kidney (HK) and red blood cells (RBC) homogenates] became more available. 

Initially, the screening was done using Chilean (PRV-1-unknown subtype) HK homogenate on 11 cells lines using the standard supernatant transfer sub-cultivation protocol recommended by the OIE Manual of Diagnostic Tests for Aquatic Animals for isolation of disease causing viruses, such as viral haemorrhagic septicaemia virus, infectious haematopoietic necrosis virus, and infectious salmon anaemia virus. Additionally, a long-term screening experiment was done with the ASimf20 cell line exposed to Chilean (PRV-1-unknown subtype) HK homogenate. This is a single cell culture passage isolation assay, but instead of terminating early, the exposed cells were kept alive with weekly fresh medium changes for up to 3 months. Replicate cultures were collected at 2 h (to determine initial Ct), 1 month, and 3 months for analysis by RT-qPCR. Full description of results for the initial screening experiment is described in Section 2.1.1 below and presented in Table 2. Full description of results for the long-term screening experiment is described in Section 2.1.3.

Subsequently, screening of 26 cell lines for capacity to support Canadian (PRV-1a) and Norwegian (PRV-1b) replication and amplification was performed but more comprehensively, using up to five virus isolation assays. This increased screening rigor is due to the lack of PRV-1 amplification from the initial screening of cell lines with the Chilean (PRV-1-unknown subtype) HK homogenate briefly mentioned above. The virus isolation assays (described in detail in the Materials and Methods section) were: (1) single cell culture passage, (2) sub-cultivation of exposed cells with fresh cells, (3) sub-cultivation of only exposed cells, (4) cell lysate transfer, and (5) supernatant transfer. At least one virus isolation method was examined in each cell line, and for some cell lines, all five virus isolation methods were attempted. The homogenate source for Canadian PRV-1a and Norwegian PRV-1b experiments was RBCs from infected fish. Full description of results for screening Canadian (PRV-1a) and Norwegian (PRV-1b) amplification is described in Section 2.1.2 below and presented in Table 3.

In addition to monitoring for PRV-1 replication by RT-qPCR in the above screening experiments, the potential appearance of CPE in the inoculated cell lines was also monitored and will be described in Section 2.1.3 below.

#### 2.1.1. Lack of PRV-1 Amplification in 11 Cell Lines Exposed to Chilean PRV-1 (Unknown Subtype) 

A total of 11 cell lines from five fish species were tested for capacity to support PRV-1 amplification upon exposure to Chilean (PRV-1 unknown subtype) HK homogenate. In these experiments, the supernatant transfer virus isolation assay was used (assay described in detail in the Materials and Methods section). Specifically, for these cell lines, supernatant was transferred (passaged) to new cell cultures in a serial manner at day 7, 14, 21, and 28, for a total of four passages and seven days between each passage. Cell samples were collected for RT-qPCR analysis at the same time points. For RTL-W1, PRV-1 was not detected at any time point, as the cycle threshold was not crossed (Table 2). For RTHDF, PRV-1 was not detected in three of four times points, and in the day 14 (passage 2) time point positive for PRV-1, only one of three biological replicates was positive. This could be due to cross-contamination during the RT-qPCR assay. For RTG-2, PRV-1 was not detected at day 7 (passage 1) or 14 (passage 2), faintly detected at day 21 (passage 3), and faintly detected at day 28 (passage 4) in only one of three biological replicates. For all the remaining cell lines, PRV-1 was detected in the first passage at day 7, with Ct values ranging from 27.3 to 33.7; however, in subsequent days and passages, the Ct values either increased relative to day 7, or were not detected. Therefore, the Chilean (PRV-1-unknown subtype) strain did not replicate consistently and continually in the cell lines examined.

#### 2.1.2. Lack of PRV-1 Amplification in 26 Cell Lines Belonging to 13 Species Exposed to Canadian and Norwegian PRV-1 RBC Homogenates

To determine whether PRV-1 replicated and amplified in 26 cell lines, the Ct value of samples collected at the final time point was compared against the Ct value of samples collected at the initial time point. In virus isolation assay with only one passage, the initial time point is during an early day (day 2) post-inoculation, and the final time point is day 14 post-inoculation. In virus isolation assays with multiple passages, the initial time point is at the end of the first infection passage and the final time point at the end of the second passage (Table 3). In most of the 26 cell lines examined, the Ct value at the final time point was either higher than the initial time point, within 0.5 Ct if lower, or not detected; all three outcomes are considered as negative for amplification of PRV-1 RNA (Table 3). The exceptions are described as follows. For PBLE, in the single cell culture passage isolation assay, the final Ct was lower than the initial Ct but in only one of two biological replicates. The Ct value in the second replicate was undetectable. For ASCF, in the sub-cultivation of only exposed cells assay for the Canadian PRV-1a, the final Ct was lower than initial Ct, but the difference was less than 1.0. The same experiments performed with the Norwegian PRV-1b resulted in a higher final Ct value. For ASHe, in the single cell culture passage isolation assay for the Norwegian PRV-1b, the final Ct value at day 14 was lower than the initial Ct by 1.4, but the final Ct value was still high at 32.1. For RTgutGC, RTHDF, RTH-149, and STB5 Gill in the single cell culture passage isolation assay for Canadian PRV-1a, the final Ct values were lower than initial Ct by a range of 1.0 to 3.0, indicating potential low to moderate level of PRV-1 amplification within the first passage. However, this was not the case in the same experiments performed with Norwegian PRV-1b. Additionally, for RTgutGC, RTHDF, and RTH-149, other assays that involved multiple passages showed no PRV-1 amplification. For GarL, in the single cell culture passage isolation assay for both the Canadian PRV-1a and Norwegian PRV-1b, the final Ct values of 27.6 and 30.2 were 2.1 and 3.0 Ct lower than initial Ct values, respectively. This indicates potential moderate level of PRV-1 amplification in the first passage, but additional experiments involving multiple passages are required to confirm consistent PRV-1 amplification. This case could be like the previously discussed cell lines, where, upon multiple passages, the Ct value increased or became undetectable. Overall, PRV-1 did not amplify in most cell lines examined, regardless of virus isolation method. While the single cell culture passage isolation assay experiment suggests potential low level Canadian PRV-1a amplification within the first passage in some cell lines, this replication did not continue in experiments involving multiple passages.

#### 2.1.3. Lack of Consistent Cytopathic Effects (CPE) in Cell Lines Screened for PRV-1 Amplification 

**Figure 2:**
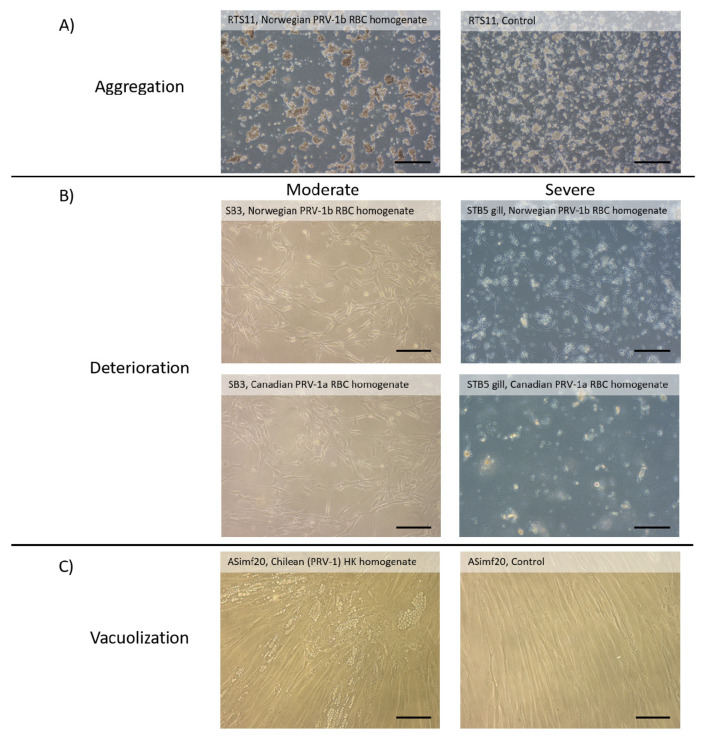
Rare changes in culture appearance after exposure to PRV-1 homogenates. Although for the vast majority of cultures no change was seen as viewed by phase contrast microscopy, occasionally changes were noted with some cell lines and homogenates. (**A**) Aggregation of the monocyte/macrophage RTS11 cells over 2 weeks in control cultures (right photo) and the slightly more pronounced aggregation after exposure to PRV-1 RBC homogenates from Norwegian fish (photo on left). (**B**) Moderate (left side photos) and severe (right side photos) deterioration in the monolayer of cell lines from the sturgeon brain (SB3) and threespined stickleback gill (STB5 gill) two weeks after exposure to either Norwegian PRV-1b (top photos) or Canadian PRV-1a (bottom photos) RBC homogenates. (**C**) Appearance of vacuoles in cultures of the Atlantic salmon intestinal myofibroblast cell line ASimf20 eight weeks after exposure to AS PRV-1 head kidney homogenates from Chile (left side photo) but not in control cultures (right side photo). Scale bar represents 200 microns.

Cell lines screened with either Chilean (PRV-1 unknown subtype), Canadian (PRV-1a), or Norwegian (PRV-1b) in screening experiments described above were regularly monitored for appearance of CPE. As viewed by phase contrast microscopy, the appearance of cell cultures with control and PRV-1 homogenates occasionally changed, but any change occurred erratically, with no consistent association with viral homogenates. In cultures of the monocyte/macrophage cell line, RTS11, some cells are also in suspension and others are loosely adherent on the flask surface. With homogenates, floating RTS11 cells formed clumps (Figure 2A). All the other cell lines were adherent and incubated with the homogenates as monolayers. In nearly all these cultures, the cells kept either their epithelial-like or fibroblast-like shapes and their organization as sheets of attached cells. Few exceptions were seen where the monolayer appeared to deteriorate over time in homogenates and the level of deterioration ranged from moderate to severe (Figure 2A). Finally, vacuoles appeared occasionally in cultures of a few cell lines with PRV-1 homogenates and also in control cultures, although less frequently. The most pronounced example of vacuolization was in long-term infection of ASimf20 with Chilean (PRV-1 unknown subtype) HK homogenates (Figure 2C). However, the average Ct values for PRV-1 in these long-term cultures were 32.9 at month one and 34.0 at month three, suggesting the presence of vacuoles is not a sign of PRV-1 amplification. Overall, CPE was not a useful endpoint in the search for cell lines supportive of PRV-1 production.

### 2.2. Lack of PRV Amplification in Cell Lines Co-Administered with Either Multiple PRV-1 Isolates, PRV-1 and CSV, or PRV-1 and IPNV 

Three co-administration experiments of cell lines were performed to determine if the properties of these other viruses can enhance PRV-1 replication, since cell lines exposed to individual PRV-1 isolates did not promote PRV-1 amplification in the previous screening experiments. First, cell lines were co-administered with two different isolates of PRV-1, the Norwegian (PRV-1b) and Canadian (PRV-1a) isolates, with the aim of allowing for possible reassortment of PRV-1 genomes in infected cells, since the PRV-1 genome is fragmented and consists of 10 segments. Second, cell lines were co-administered with PRV-1 and CSV, with the aim of again allowing for possible reassortment, since CSV is also a reovirus, and to determine if the syncytia inducing capacity of CSV can aid PRV-1 dissemination in vitro. Third, cell lines were co-administered with PRV-1 and IPNV to determine if the lytic capacity of IPNV can promote PRV-1 dissemination in cultures. 

Co-administration of cell lines with two PRV-1 isolates was done on seven cell lines and monitored at two time points, day 2 and day 14, over one passage (Table 4). The Ct values increased in five of seven cell lines (EelB, PBLE, ASHe, ASimf20, and RTgill-W1) from day 2 post-inoculation to day 14 post-inoculation. In two cell lines, ASCF and BAASf, only a minor decrease of 0.5 Ct or lower was recorded on day 14 compared to day 2 (Table 4), suggesting co-administration of PRV-1 variants did not enhance PRV-1 amplification in vitro. 

Co-administration of cell lines with either PRV-1 and CSV or PRV-1 and IPNV was done on eight cell lines over two passages. In the first passage, samples were collected between 13 to 18 days post-inoculation, and in the second passage, samples were collected between 12 to 14 days post-passaging, which is between 26 to 32 cumulative days post-initial inoculation in passage one. In all cases, the Ct value was either higher or not detected in samples collected in the second passage (Table 5). This result suggests a lack of PRV-1 amplification, and co-administration with either CSV or IPNV was not synergistic for PRV-1 amplification. Cytopathic effects were observed in these co-administrations. For the co-administration with CSV, syncytia and considerable cell death were observed in both passages for CHSE-214. Cell death was observed in passage 1 for EPC and RTgill-W1 but reduced in passage 2. Homotypic aggregation of cells was observed in RTS11 in passage 1 but not in passage 2. For the co-administration with IPNV, cell lysis of varying severity was observed in four cell lines. In ASHe, complete cell death and destruction of the monolayer occurred. In BAASf and RTgill-W1, severe monolayer destruction occurred but some viable cells remained. In CHSE-214, some cell death was observed but many viable cells remained. For RTS11, as was the case in the co-administration with CSV, homotypic aggregation was observed in passage 1 but not in passage 2. The CPE from both CSV and IPNV co-administration confirmed the impact of these viruses on the cell lines but their presence did not promote PRV-1 amplification.

### 2.3. PRV-1 RBC Homogenates Induce Mx Protein Expression in Atlantic salmon and Rainbow Trout Cells Lines

**Figure 3:**
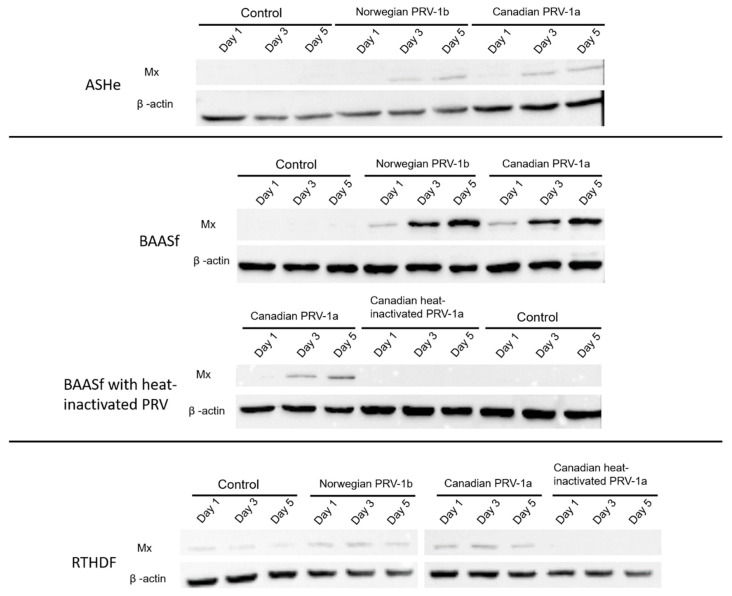
Induction of Mx in salmonid cell lines after exposure to PRV-1 RBC homogenates. Cultures of the Atlantic salmon heart endothelial (ASHe) cell line, bulbus arteriosus of Atlantic salmon fibroblast (BAASf) cell line, and rainbow trout hypodermal fibroblast (RTHDF) cell line were exposed to either Norwegian (PRV-1b) or Canadian (PRV-1a) RBC homogenates and examined for Mx proteins expression by Western blot on days 1, 3, and 5. BAASf and RTHDF was also exposed to heat-inactivated Canadian (PRV-1a) RBC homogenates. In all three cell lines, Mx proteins were upregulated by exposure to homogenates containing either PRV-1 subtypes. Mx proteins levels in cells exposed to control homogenates was either lower or not detectable when compared to levels in cells exposed to PRV-1 homogenates. Similarly, Mx proteins levels in cells exposed to heat-inactivated Canadian PRV-1a homogenate was not detectable.

The interaction between PRV-1 homogenates and cell lines has been described using only two observational indicators so far, the changes in Ct values and CPE; therefore, the potential induction of an antiviral response in cell lines by PRV-1 homogenates was used as a third indicator of interaction. Three salmonid cell lines (two from Atlantic salmon and one from rainbow trout) were examined. In this case, only salmonid cell lines were chosen because the anti-Mx antibody was developed from rainbow trout and has known cross-reactivity with Atlantic salmon Mx but unknown cross-reactivity with other fish species. The cell lines were ASHe and BAASf from Atlantic salmon and RTHDF from rainbow trout. In all three cell lines, both the Norwegian (PRV-1b) and Canadian (PRV-1a) RBC homogenates induced Mx protein expression at levels that were higher than control RBC homogenates from uninfected fish (Figure 3). Furthermore, heat inactivation of the Canadian (PRV-1a) RBC homogenate abolished the Mx protein expression in BAASf and RTHDF (not done in ASHe) (Figure 3). These results suggest that either PRV-1 in the homogenate or antiviral factors, originally released from infected fish in response to PRV-1 infection, caused upregulation of the antiviral response in these cell lines.

### 2.4. Attempts to Establish Cell Lines from Infected Atlantic salmon Resulted in Limited Success but Showed Persistence of PRV-1 RNA in One Culture over Long Periods 

Thus far, exposure of previously established cell lines to three PRV-1 isolates resulted in no dramatic amplification of PRV-1, particularly over multiple passages and regardless of the virus isolation assay used. Since PRV-1 cannot be amplified when exposed to the currently available cell lines examined in this work, attempts were made to develop new cell lines from fish already infected with PRV-1, with hopes of successfully obtaining new cell lines already or persistently infected with PRV-1. To accomplish this task, two in vivo infection challenges were performed to produce PRV-1-infected Atlantic salmon. The first infection challenge was done with Canadian PRV-1a at PBS-DFO in Nanaimo, B.C., and the second was done with Chilean PRV-1 (unknown subtype) at a level 3 facility that previously belonged to Elanco Canada in Victoria, P.E.I. The results of these two attempts are described in detail below. Since the end goal is to obtain permanent cell lines, PRV-1 screening was not performed on the initial individual primary culture explants, as this would interfere with cell growth and reduce the potential and probability for these primary cultures to develop into permanent cell lines downstream. However, PRV-1 was confirmed systemically in the fish used to develop primary cultures.

For the experimental infection trial with PRV-1a at PBS-DFO in Nanaimo, B.C., infected fish produced high levels of PRV-1a (Supplementary Table S1). Week 2 and week 3 post-inoculated fish produced most virus, which reduced by week 4. To increase the chances of obtaining permanent cell lines, numerous primary cultures explants were initiated as part of a brute-force strategy. In total, primary cultures were initiated from 30 hearts, 30 spleens, and 30 head kidneys tissues for each of the 30 PRV-1a infected fish. Cultures were successfully initiated, defined as visible attachment or growth of cells, in many flasks of all tissue types, but over time only some of the heart and spleen cultures retained at least one field of view of cells after the first sub-cultivation. These cultures were kept for approximately 30 months post-initiation, but by 30 months, most had few to no remaining viable cell outgrowth and were discarded. Unfortunately, no permanent cell lines arose from any of the initial primary culture explants. However, remnant tissue fragments of various sizes from the original explants were still visible in some of the remaining primary cultures. Two of these cultures (one heart and one spleen) contained sufficient tissue amounts and were collected for PRV-1 screening by RT-qPCR. The heart culture showed no detectable PRV-1 RNA (no Ct value) but the spleen culture showed a Ct value of 31.4. While the initial Ct values in these primary cultures at the time of explant (~30 months prior) were not determined (in order to keep as many cultures available to increase the chances of obtaining permanent cell lines), this result suggests that although not necessarily amplifying or infectious, PRV-1 RNA can remain detectable in vitro for long periods.

For the experimental infection trial with Chilean PRV-1 (unknown subtype) at Elanco Canada’s previously owned level 3 facility, Atlantic salmon were successfully infected with PRV-1 but the amount of virus in fish was low at both week 2 and week 3 post-infection (Supplementary Table S1). Primary cultures were initiated from 18 spleens and 18 head kidneys for each of the 18 PRV-1 infected fish as part of a brute-force strategy. Of these, only one head kidney primary culture developed into a permanent cell line, name HK15. This cell line has been grown for over 10 passages. However, screening of passage 7 and 8 for PRV-1 showed a lack of PRV-1 presence. The two replicates screened in passage 7 and passage 8 showed Ct values of ≥ 35.0. Since the initial Ct values in the initial primary culture explants (~30 months prior) were not determined in order to keep the cultures growing potentially to become cell lines, this result suggests two possible explanations: either the head kidney of this fish did not originally contain PRV-1, or it did contain PRV-1 but PRV-1 was not maintained in the resulting cell line. For similar reasons, Ct values of earlier passages of this newly developing cell line were not determined. Cells from earlier passages were kept growing and expanding in order to reduce the risk of culture loss.

## 3. Discussion

Cell line cultures from 14 fish species and multiple organs were exposed in different ways and conditions to RBC and kidney homogenates from AS with PRV-1 and examined for up to 3 months for changes in appearance and PRV-1 Ct values. These were largely unchanged, indicating that the cell lines did not support PRV-1 amplification. In the context of Figure 1, the 31 cell lines are argued not to belong to either the lytically permissive or persistent PRV-1 invitromes, but instead are in the non-supportive invitrome, which has importance in virology as a framework for delineating cell and host tropism and for studying the interactions between infected and bystander cells.

None of the cultures exposed to PRV-1 homogenates acquired the defining properties of lytically permissive cell cultures: viral replication, significant culture deterioration, and capacity to transfer or passage to new cultures the same properties. On a few occasions, substantial monolayer deterioration was seen, with one example occurring with sturgeon brain SB3, but this was not consistently found and cannot be attributed to PRV-1 because over the time of monolayer deterioration, Ct values were unchanged. One possibility is that the SB3 cells were responding to another virus. However, this is unlikely to be a known salmon virus, such as IPNV, IHNV, VHSV, or CSV, because the same homogenates elicited no CPE in CHSE-214, EPC, and RTG-2, which are cell lines known to detect them [15]. Also, a new and yet to be described virus is an unlikely cause because the CPE was not transmittable to new cultures. More likely, the homogenate caused SB3 cultures to deteriorate for non-viral reasons. In older literature, mammalian organ extracts were sometimes found to have cytotoxic and cytostatic factors [34,35,36,37]. Their identity has never been completely resolved but fatty acids like arachidonic acid are candidates [38] and are possibly acting here.

The occasional vacuolization of cultures after exposure to PRV-1 homogenates hints at the possibility that some cell lines might be partially permissive to PRV-1, with the vacuoles being the visible indicator (CPE) of infection. Previously, after exposure to PRV or HSMI homogenates, cultures of ASK, GF-1, EPC, and FHM were reported to become vacuolated, but in all these cases vacuolization disappeared with passaging [9,10,11]. Additionally, vacuolization was not consistently seen in all cultures [9], which also was the experience in the current study as best illustrated with ASimf20. On occasion, monolayers of this cell line developed prominent regions of vacuolization several weeks after exposure to PRV-1 homogenates but at other times, the control cultures did as well. Spontaneous vacuolization of animal cell lines has long been observed, but the exact cause in most cases has been unclear [39]. Possible contributing factors are the buildup of ammonia in cultures over several weeks without a medium change [40], or the presence in homogenates of ammonium from RNALater [41] or of the antifungal agent, amphotericin [42]. A possible connection between PRV-1 and vacuolization in vitro should be investigated further because vacuoles have been seen in skeletal muscle of PRV-infected fish [43].

Whether some cell lines could be persistently infected is interesting to consider. Recently, AS were described with a persistent, low-activity but productive PRV infection, and PRV RNA persisted in erythroid progenitor cells and macrophages, especially in the kidney but also in the spleen [44]. In the current study, primary cell cultures were initiated from two batches of PRV-infected fish but only one culture led to a cell line: head kidney 15. HK15 cultures without prior exposure to PRV-1 homogenates always had PRV-1 RNA Ct values over 35.0, which rules out the cell line being persistently infected with PRV-1. This deserves more attempts, and with a focus on primary long-term hemopoietic cultures (LTHC) of the kidney and spleen [45], which was the route to the monocyte/macrophage cell line RTS11 [46]. However, for the cell lines of the current study, including for RTS11, the Ct values did not follow the pattern expected for either chronic diffuse or latent cultures where each cell will have the virus and, as cells accumulate over time, so will viral RNA, which should lead to a drop in Ct values rather than no change or an increase in Ct values. More difficult to rule out are chronic focal cultures where only a small percentage of cells become infected, produce a virus, and possibly die. In this case, the amount of viral RNA produced in cultures might be so small that changes in Ct values are missed. Similarly, any CPE, cytolytic or otherwise, might occur in so few cells that overall culture appearance seems unchanged. PRV appears to have the potential to be cytolytic because when ectopically expressed, the PRV protein, p13, was found to be cytotoxic [47] but both in vitro and in vivo observations suggest that any cytolysis is at a very low level [8,32,48,49,50]. Judging whether a low level of cytolysis occurred in RTS11 cultures was made difficult by the cells aggregating in suspension, which appeared to be enhanced by PRV-1 homogenates. The aggregates could represent homotypic aggregation [51] or clumping by debris from a few dying cells. Thus, chronic focal cultures seem unlikely but difficult to completely rule out.

With the other possibilities being unlikely, the cell lines of this study are concluded to be in the non-supportive PRV invitrome. In the future, several research paths can be taken to determine whether this invulnerability is a fundamental property of the cells or caused by experimental inadequacies. For example, the cell lines might be screened with PRV purified from RBCs to make sure that something in the homogenates has not interfered with infection, and also to be able to test at a high multiplicity of infection, which might allow cellular antiviral mechanisms to be overwhelmed [52]. Additionally, more sensitive endpoints for monitoring the PRV life cycle might, in future, reveal that some non-supportive cell lines are in fact partially supportive. Despite these qualifications, identifying a large group of cell lines as being non-supportive to a particular virus is important for at least two general reasons. Firstly, patterns might emerge that yield insight into the tropism of the virus at the cellular and host levels and give direction to future searches for supportive cell lines. Additionally, the cell lines can be used to help identify the factors responsible for restricting infections to particular cell types and hosts. Secondly, the cell lines can be used to study bystander cells in hosts.

PRV appears to have a rather narrow cellular tropism, as judged by the fact that cell lines from multiple tissues and organs were found to be non-supportive and by the histological examination of others on PRV-infected fish that showed restricted localization of PRV RNA and polypeptides. RTS11 from the spleen and ASHe from the heart represented macrophage and endothelial cells respectively, whereas the other cell lines provided homogeneous cell populations of either fibroblast or epithelial cells from a wide range of anatomical sites, including brain, gut, heart, liver, and skin. Presumably, the cell lines lack specific receptors or internalization capabilities for PRV-1 entry and/or intracellular components for PRV-1 gene expression. In PRV-infected fish, susceptible cell types appear to be erythroid progenitors and possibly cardiomyocytes and hepatocytes [43,44]. Inasmuch as no success was achieved with individual cell lines and because detection of some mammalian viruses was enhanced by mixing cell types [53,54], mixed cultures were tried (data not shown). These were cultures of an epithelial cell line with two fibroblast cell lines from different anatomical sites in the same species, which were either AS or walleye, or of three rainbow trout epithelial cell lines from the liver, gill and gut. Again, PRV-1 amplification was not supported (data not shown).

From the perspective of possible hosts, the results suggest that surveying cell lines from more fish species seems unlikely to uncover PRV-1 supportive cell lines. No supportive cell lines were found in invitromes from 14 species of ray-finned fish (Class Actinoptergyii), representing a wide range of taxonomies, life histories, and geographies. The range is wider than the range of species in which PRV has been detected. To date, PRV has been found in salmonids and in 4 marine species, capelin *Mallotus villosus*, Atlantic horse mackerel *Trachurus trachurus*, Atlantic herring *Clupea harenugs* and great silver smelt *Argenita silus* [8,55]. For the cell lines, the species include one from the subclass Chondrostei, the Lake sturgeon *(Acipenser fulvescens)*, with the rest from the subclass Neopterygii, including one from the infraclass, Holostei, longnose gar (*Lepisosteus osseus*). The salmonid invitrome was the most comprehensively investigated family, with four species: AS (*Salmo salar*), rainbow trout (*Oncorhynchus mykiss*), Chinook salmon (*O. tshawytscha*), and Arctic char (*Salvelinus alpinus*). For life histories, five species were anadromous: AS, Rainbow trout, Chinook salmon, Arctic char, and the threespine stickleback (*Gasterosteus aculeatus*). One was catadromous, the American eel (*Anguilla rostrata*). Six species were from freshwater: Lake sturgeon, Longnose gar, Fathead minnow (*Pimephales promelas*), Walleye (*Sander vitreus*), Yellow perch (*Perca flavescens*) and Zebrafish (*Danio rerio*). The two marine species were Pacific herring (*C. pallasii*) and haddock *Melanogrammus aeglefinus*). Geographically, five species were from the North American Great Lakes basin: Fathead minnow, Lake sturgeon, Longnose gar, Walleye, and Yellow perch. Three species (Chinook salmon, rainbow trout and threespined stickleback) were from the Northeast Pacific and three (AS, haddock, and American eel) from the North Atlantic. Finally, the natural geographical locations of the Arctic char and zebrafish are, respectively, the Arctic ocean and South Asian freshwater.

One potential use of non-supportive cell lines is as surrogates for bystander cells in order to study in vitro how bystanders of different anatomical and tissue origins interact with and respond to virus-infected cells. In PRV-infected fish, the RBCs and their progenitors are susceptible [1,44,56] and possibly cardiomyocytes and hepatocytes [43], while based on current information, the other cell types might be regarded as bystanders. The infected RBCs and bystanders would not interact by forming intercellular junctions but would be expected to get close, especially in clots. As well as producing virions, RBCs can produce cytokines/interferons [49,57,58] and activate antiviral mechanisms [58]. In the current study, a few cell lines were evaluated for the induction of the antiviral protein Mx after exposure to PRV-1 RBC homogenates. Strong induction was seen in Atlantic salmon and rainbow trout cell lines but not if the homogenates had been heated, hinting at the involvement of cytokines/interferons. This can be tested in the future by comparing Mx induction in these cell lines with purified PRV versus the medium from PRV-infected RBC cultures. In PRV-infected fish, Mx transcripts were induced in the heart and blood cells [8,10,59] but the response in the head kidney and liver was less clear [8], suggesting that bystander responses vary between tissues and/or organs. This can be tested in the future by evaluating Mx induction by PRV-RBC homogenates with a wider range of salmonid cell lines.

In conclusion, no fish cell lines from a range of organs and species were found that supported the amplification of PRV-1 from Atlantic salmon homogenates, as evidenced by no consistent change in the appearance and no consistent decrease in Ct values of cell cultures. These cell lines are referred to as the non-supportive PRV-1 invitrome and should be useful for studying the basis of the cellular and host tropism of PRV-1, and for understanding bystander interactions with infected cells. Future searches for supportive lines should focus on the erythroblast lineage.

## 4. Materials and Methods 

### 4.1. Cell Line Maintenance 

The cell lines are provided in Table 1. Briefly, all cell lines were propagated using L15 medium (L15) with fetal bovine serum (FBS) concentration ranging from 10% to 20% supplemented with 1% penicillin-streptomycin (PS, growth medium). For some early passage cultures that were developed from primary culture, 30% FBS concentration was used. The general steps for propagation of cell lines is as follows. Old medium was removed from flasks containing cells and the cell monolayer was washed 1x with Dulbecco’s phosphate-buffered saline (DPBS), 1 mL for T12.5 and T25 flasks and 3 mL for T75 flasks. After rinsing, trypsin-EDTA (0.125%) was added to detach cells at the same volumes as stated above for the washing step. After cells detached, enough growth medium was added to the cell and trypsin-EDTA mixture to satisfy the propagation (or passaging or “split”) ratio and total growth volume in each flask. The final volume of growth medium per flask ranges from 12 to 15 mL for T75 flasks, 4 to 6 mL for T25 flasks, and 2 to 4 mL for T12.5 flasks. The growth temperature range 20–22 °C. The time between propagation was typically 2 to 3 weeks, and passaging ratio for each cell line was typically between 1:2 and 1:4.

### 4.2. PRV-1 Inoculum Preparation 

PRV-1 inoculums were prepared from either head kidney tissues or whole blood. Head kidney tissues were aseptically harvested from farmed Atlantic salmon in Chile that exhibited clinical signs and mortality consistent with HSMI, based on histopathology and qPCR analyses, after transfer into a freshwater facility. The kidneys were homogenized using a Dounce homogenizer and sonicated for one minute in intervals of 10 s at 30% intensity. Homogenized samples were centrifuged for 10 min and the pellets were suspended in Minimum Essential Medium (MEM) media with the same volume as the removed supernatant.

Whole blood was sourced from Atlantic salmon experimentally infected with PRV-1. Blood was collected from the caudle vein of infected fish via 22-gauge needles and syringes, and immediately transferred to heparinized vacutainer tubes. The blood was stored frozen as either whole blood or blood in RNAlater. RNAlater was not removed from the mixture because it could contain materials released from lysed blood cells after thawing. Blood was homogenized by diluting the blood/RNAlater mixture 1 to 10 in L15 containing antibiotics and homogenized with a dounce homogenizer. The tubes were spun at 2000× *g* for 5 min at 4 °C. Plasma was removed and cell pellet suspended in 10× (of original blood volume) L15 supplemented with 50 μg/mL gentamicin (GS). For example, if original blood volume was 1 mL then added 10 mL of L15 to the pellet. Suspended pellet was sonicated with a Branson Sonifier (Branson Ultrasonics Corp, Danbury, CT, USA) on ice for 1 min and 20 s in 10 s bursts with 30 s rests and then spun at 2000× *g* for 5 min at 4 °C to pellet cellular debris. The clarified supernatant was kept for use as inoculum. Whole blood inoculum was also prepared from a few Atlantic salmon that had no history of exposure to PRV-1 or HSMI homogenates and had negative Ct values for PRV-1. These are referred to as control homogenates.

### 4.3. Primary Culture Originating from PRV-1 Infected Atlantic salmon 

Two experimental infection trials were performed to infect Atlantic salmon with PRV-1a and PRV-1 (unknown genotype) and to develop cell cultures from the infected fish. The trials were at Fisheries and Oceans Canada, Pacific Biological Station (PBS-DFO; trial one) and a level 3 R&D facility in Victoria, PE (trial two) that previously belonged to Elanco Canada. The experimental protocols varied between the two trials and are described in detail below. The PRV-1 RNA level (Ct values) for the infected fish is listed in Supplementary Table S1. The infection of fish and subsequent primary culture initiation procedures are described below.

In trial #1, Atlantic salmon (approximately 50 g weight) held in 10–12 °C brackish water (10 ppm) were anesthetized with MS-222 and injected with 100 μL of blood PRV-1a inoculum as described previously [2]. After injection, fish were held in 30 ppm seawater at 10–12 °C. At each of two, three, and four weeks post-infection, ten fish were euthanized with an overdose of MS-222, and heart, head kidney, and spleen tissues were aseptically dissected for establishment of primary cultures. The growth media used to initiate primary culture was L15 medium supplemented with 1% PS, 1% Amp. B, 0.1% GS and 30% heat-inactivated FBS. The wash solution used was DPBS supplemented with 1% PS, 1% Amp. B, 0.1% GS. Primary cultures were initiated from two-week infected fish by explant outgrowth. Tissues were minced into sizes ranging from 1 to 3 mm^2^, washed once, then transferred into T25 flasks, with each flask containing 1 mL of growth medium and approximately 12 pieces of minced tissue. For the week three infected fish, primary culture was initiated using 500 μg/mL of either collagenase type II or IV digestion. Tissues were minced into sizes ranging from 1 to 3 mm^2^. Heart tissues were transferred into micro-centrifuge tubes containing 1 mL of collagenase type II. The same was done for spleen and head kidney tissues, except with collagenase type IV. Tissues were incubated overnight at 4 °C to allow for digestion. The next day, tubes were centrifuged at 1000× *g* for 5 min to pellet cells and tissues. Tissues were then suspended in 1 mL of growth medium and added to T25 flasks. Flasks were incubated at 15 °C. For the week four infected fish, primary cultures were initiated using trypsin digestion. Tissues were minced into sizes ranging from 1 to 3 mm^2^ and incubated in trypsin overnight at 4 °C. After digestion, the transfer of tissues into T25 flasks was done as described for the collagenase treated tissues.

In trial #2, Atlantic salmon (approximately 147 g weight) were infected by intraperitoneal injection of 100 μL of Chilean head kidney tissue homogenate containing PRV-1 (unknown subtype). Fish were held in a 400 L circular tank, with UV filtered saltwater, in a flow-through system, with a salinity range 29.9–34.5 ‰, and at a temperature range of 10.0–12.4 °C for 21 days post-exposure. At two- and three-weeks post-infection, ten fish were euthanized with an overdose of MS-222, and head kidney and spleen tissues were collected for establishment of primary cultures. The growth media used to initiate primary culture was L15 medium supplemented with 1% PS, 1% Amp. B, 0.1% GS, and 20% FBS. The wash solution used was DPBS supplemented with 1% PS, 1% Amp. B, and 0.1% GS. On the day of primary culture initiation, head kidneys and spleens were minced into sizes ranging from 1 to 3 mm^2^ and added to T12.5 flasks containing 0.5 mL of growth medium. All flasks were incubated at 20 °C. The next day, 4 mL of growth medium was added to each flask and the cultures were allowed to develop.

### 4.4. Monitoring for PRV-1 Amplification in Established Cell Lines Using Five Virus Isolation Assays 

PRV-1 replication in cell lines was evaluated and monitored using five different virus isolation assays. Detailed methods of the assays are provided in their independent subsections below. One assay involved only a single cell culture passage (single passage isolation). The other four virus isolation assays involved at least two cell culture passages. They are termed: sub-cultivation of exposed cells with fresh cells, sub-cultivation of only exposed cells, cell lysate transfer, and supernatant transfer.

#### 4.4.1. Monitoring for PRV-1 Amplification Using Single Cell Culture Passage Isolation Assay 

In single cell culture passage isolation assays, cell lines were exposed to PRV-1 inoculums, then PRV-1 replication was monitored early at day 2 post-inoculation and late at day 14 post-inoculation by RT-qPCR. Replicate inoculations were done where some of the replicates were collected at day 2, and then the remaining replicates were collected at day 14 for RT-qPCR analysis. Detailed experimental methods are described below. 

Confluent cell cultures in T25 flasks were exposed to 4 mL of Canadian PRV-1a RBC homogenate (1:500 in 2% FBS/L15) or Norwegian PRV-1b RBC homogenate (1:500 in 2% FBS/L15) continuously for up to 2 weeks. The cultures were incubated at 14 °C. Samples were collected for RNA extraction at 2 d, and then at 14 d post-exposure.

#### 4.4.2. Monitoring for PRV-1 Amplification Using Sub-Cultivation of Exposed Cells with Fresh Cells 

In sub-cultivation of exposed cells with fresh cells assays, cell lines were exposed to PRV-1 inoculums for 14 d (passage 1), then at day 14, 20% of cells were collected as viable cells and added to a new culture vessel containing a monolayer of fresh cells (passage 2). The remaining 80% of passage 1 cells were collected for RT-qPCR analysis. Fourteen days after the beginning of passage 2, all cells in passage 2 were collected for RT-qPCR analysis. Detailed experimental methods are described below. 

Confluent cell cultures in T25 flasks were exposed to 4 mL of Canadian PRV-1a RBC homogenate (1:100 in 2% FBS/L15) continuously for up to 2 weeks (passage 1). The cultures were incubated at 14 °C. At 14 d post-exposure, cells were collected using ~0.125% trypsin, and 20% of collected cells (diluted in 4 mL of 2% FBS/L15) were added to a new flask containing a monolayer of fresh cells (passage 2). The remaining 80% of passage 1 cells were collected for RT-qPCR analysis. At 14 days into passage 2, all cells were collected for RT-qPCR analysis.

#### 4.4.3. Monitoring for PRV-1 Amplification Using Sub-Cultivation of Only Exposed Cells 

In sub-cultivation of only exposed cells assays, cell lines were exposed to PRV-1 inoculums for 14–22 d (passage 1) depending on the cell line, then at that day, half of the cells were collected as viable cells and added to a new culture vessel (passage 2). The remaining half of passage 1 were collected for RT-qPCR analysis. From 21 to 30 d (depending on the cell line) after the beginning of passage 2, all cells in passage 2 were collected for RT-qPCR analysis. Detailed experimental methods are described below. 

Confluent cell cultures in T25 flasks were exposed to 4 mL of Canadian PRV-1a RBC homogenate (1:100 to 1:160 dilution in 15% FBS/L15) or Norwegian PRV-1b RBC homogenate (1:80 to 1:100 dilution in 15% FBS/L15) continuously for up to 22 d (passage 1). High FBS concentration was used because the cells were being propagated. After 14–22 days (depending on the cell line), old medium was then removed, and the cells were washed once with 2 mL DPBS. One mL of ~0.125% trypsin was added to detach cells. After cellular detachment, 1 mL of 15% FBS/L15 was added to inactivate trypsin and mix cells. Half the cells (1 mL) was collected for RNA extraction and the second half was mixed with 3 mL of 15% FBS/L15 and added to a new T25 flask to propagate in passage 2. For this part of the experiment, homogenate availability was limited at the time, so this resulted in variable dilutions of PRV-1 homogenate used throughout this period.

#### 4.4.4. Monitoring for PRV-1 Amplification Using Cell Lysate Transfer 

In cell lysate transfer assays, cell lines were exposed to PRV-1 inoculums for 14 days (passage 1), then, at day 14, cells were lysed using water and freeze-thaw lysis. A portion of the lysate was added to a new culture vessel containing a monolayer of fresh cells (passage 2). The remaining cell lysates of passage 1 were collected for RT-qPCR analysis. Fourteen days after the beginning of passage 2, all cells in passage 2 were collected for RT-qPCR analysis. Detailed experimental methods are described below. 

Cell cultures in T25 flasks were exposed to Canadian PRV-1a or Norwegian PRV-1b RBC homogenate (1:100 dilutions in 4 mL of 2% FBS/L15) continuously for 14 d (passage 1). Subsequently, the supernatant was removed, and 2 mL of cell culture water was added to each flask. The flasks were incubated with water for at least 5 min before being placed at −80 °C for at least 5 min (until the liquid was frozen). The flasks were then left to thaw at room temperature and mixed by pipetting, and cellular lysis was confirmed under an inverted phase contrast microscope. After freeze-thaw lysis, 500 μL of lysate mixture was added to a new confluent flask of fresh cells containing 4.5 mL of 2% FBS/L15 (passage 2). The remaining 1.5 mL of lysate mixture was frozen at −80 °C and 200 μL of that was later used for RNA extraction.

#### 4.4.5. Monitoring for PRV-1 Amplification Using Supernatant Transfer 

The supernatant transfer assays were done with two types of PRV-1 homogenates from different tissues. One was the Canadian PRV-1a and Norwegian PRV-1b RBC homogenate, and the other was the Chilean PRV-1 (unknown subtype) head kidney homogenate. For the RBC homogenates, cell lines were exposed to PRV-1 inoculums for 14 to 26 d depending on the cell line (passage 1), then, at that day, a portion of the supernatant was collected and was added to a new culture vessel containing a monolayer of fresh cells (passage 2). All cells of passage 1 were collected for RT-qPCR analysis. Fourteen days after the beginning of passage 2, all cells in passage 2 were collected for RT-qPCR analysis. For the head kidney homogenate experiments, the protocol was similar, except that up to four passages were done for each cell line and the time between each passage (supernatant transfer) was 7 d. Detailed experimental methods are described below.

Cells were seeded into T25 flasks and allowed to become confluent or near confluent. The cultures were exposed to either 2 mL Canadian PRV-1a RBC homogenate (1:20 in 2% FBS/L15), 2 mL Norwegian PRV-1b RBC homogenate (1:20 in 2% FBS/L15), or 2 mL Chilean PRV-1 (unknown subtype) head kidney homogenate (1:10 in 2% FBS/L15). Cultures were incubated overnight before the inoculums were removed and cell monolayer washed once with 2 mL DPBS. Four mL of fresh 2% FBS/L15 was then added to each flasks and flasks were incubated at 14 °C for 14 to 26 d (for RBC homogenates) and 7 d (for head kidney homogenates) before supernatant was collected to inoculated fresh cells in passage 2 and cells were collected for RT-qPCR analysis. After 14 d (for RBC homogenates) and 7 d (for head kidney homogenates) of incubation in passage 2, cells were collected for RT-qPCR analysis. At this point, the experiments with RBC homogenates ended, but the experiments with head kidney homogenates continued for two additional passages up to passage four.

#### 4.4.6. Assessing the Appearance of Fish Cell Cultures upon Exposure to PRV-1 Homogenates 

For the possibility that cytopathic effect (CPE) would develop, primary and cell line cultures were observed by phase-contrast microscopy shortly after the addition of PRV-1 homogenates in all infection experiments described and for the duration of those experiments. The longest observation period was for 3 months.

### 4.5. Co-Administration of Cell Cultures with Two Isolates of PRV-1, or with PRV-1 and CSV, or with PRV-1 and IPNV 

For the co-administration of cell cultures with mixed PRV-1 isolates, mixed PRV-1 inoculum was prepared by mixing one part of Canadian PRV-1a RBC homogenate (1:500 in 2% FBS/L15) with one part of Norwegian PRV-1b RBC homogenate (1:500 in 2% FBS/L15). Confluent cell cultures in T25 flasks were exposed to 4 mL of mixed PRV-1 inoculums continuously for up 14 d. The cultures were incubated at 14 °C. Samples were collected for RT-qPCR analysis at 2 d and 14 d post-exposure. 

For the co-administration of cell cultures with either PRV-1 and CSV or PRV-1 and IPNV, 10 mL of PRV-1 RBC homogenate (1:10 dilution in 2% FBS/L15) were mixed with 2 mL of either stock CSV or IPNV and 8 mL of 2% FBS/L15. This created a final mixture that contained 1:20 PRV-1 RBC homogenate with either 1:10 CSV (10^4.3^ TCID50/mL) or 1:10 IPNV (10^8.1^ TCID50/mL). Confluent cell cultures in T25 flasks were exposed to 4 mL of the virus mixture continuously for 13 to 18 d (depending on the cell line) at 14 °C (passage 1). Afterwards, the supernatant of the exposed culture was used to inoculate fresh cells in new flasks (passage 2), and cell/supernatant mixture collected for RT-qPCR analysis. Supernatant transfer assays were used in this case because CSV and IPNV was able to develop cytopathic effects in the cultures and lyse infected cells. At 12 to 14 d (depending on the cell line) after passaging, cell/supernatant mixtures were collected for RT-qPCR analysis.

### 4.6. RNA Extraction and Real-Time PCR 

RNA extraction was performed using the Qiagen RNeasy Mini Kit (Qiagen, Germantown, MD, USA) according to the manufacturer’s protocol. Following extraction, a one-step quantitative reverse transcription PCR (RT-qPCR) was used to amplify target sequences of the L1 gene in PRV [59,60]. The forward primer sequence (PRV-FWD 5′-3′) was TGCTAACACTCCAGGAGTCATTG. The reverse primer sequence (PRV-REV 5′-3′) was TGAATCCGCTGCAGATGAGTA. The hybridization probe sequence (PRV-Probe 5′-3′) was FAM-CGCCGGTAGCTCT-MGBNFQ. The PCR reaction was performed in a total volume of 10 μL, containing 100 ng of template RNA using Roche LightCycler 480 Master Hydrolysis Probes (Roche Applied Science) and following standard protocol. The cycling conditions were reverse transcription 63 °C for 3 min; denaturation, 95 °C for 30 s; amplification (37 cycles) 95 °C for 12 s, 60 °C for 45 s, and 72 °C for 1 s.

Data for PRV-1 samples in tables is shown as either a single biological replicate (average of technical replicates) or an average of two or three biological replicates. For average Ct calculations of biological replicates, if at least one of the replicates had no Ct values or Ct values greater than 35.0, then that replicate was not included in the calculation and indicated as such in the results. Samples with no Ct value or Ct values greater than 35 are labeled as “negative”. All direct Ct comparisons between cell lines and time points were conducted on the same plate (96/384 wells) to eliminate the between plate variability. Also, a standard curve was run on all plates using a serial dilution of gBlocks Gene Fragments (IDT, Coralville, IA, USA).

### 4.7. Detection of Mx2 Protein Expression in Cell Lines by SDS-Page and Western Blotting 

Three cell lines, ASHe, BAASf, and RTHDF, confluent in 6-well plates were exposed to either control, Canadian (PRV-1a), or Norwegian (PRV-1b) RBC homogenate at 1:100 dilution in 2 mL of 15%FBS/L15 per well in triplicate wells. Additionally, BAASf and RTHDF were also exposed to heat-inactivated Canadian PRV-1a RBC homogenate (1:100 dilution in 2 mL of 15%FBS/L15). Heat inactivation was done by boiling the homogenate in a water bath for 15 min. At days 1, 3, and 5 post-inoculation, protein samples were extracted using radioimmunoprecipitation assay buffer (RIPA buffer) and stored at −20 °C until used. Detailed protein extraction, protein quantification, SDS-PAGE, and Western blot procedures were done as previously described [61]. The primary antibodies were a polyclonal rabbit antibody against rainbow trout Mx proteins (1:2000 dilution in 5% skim milk/TBS-T) and a polyclonal rabbit anti-β-actin antibody (1:1000 dilution in 5% skim milk/TBS-T; Sigma-Aldrich). Both antibodies were incubated overnight at 4 °C. The secondary antibody was an HRP-conjugated goat anti-rabbit antibody (Bio-Rad) diluted to 1:5000 in 5% skim milk/TBS-T and incubated for up to three hours. Detection of protein bands was done with the SuperSignal West Pico Chemiluminescent Substrate (Thermo Fisher) and a BioRad ChemiDoc MP Imaging System (Image Lab 5.2.1 software (https://www.bio-rad.com/en-ca/product/image-lab-software?ID=KRE6P5E8Z). 

### 4.8. Ethics Approval

All work with animals was performed in strict accordance with the recommendations in the Canadian Council on Animal Care (CCAC) Guide to the Care and Use of Experimental Animals and use of live animals in experiments conducted at the PBS-DFO, were approved by the Pacific Region Animal Care Committee under protocol #16-013. The study NAH-16-025 at Elanco Canada’s R&D facility was conducted under animal use approval EIAC-0504, granted by the Elanco Animal Health Institutional Animal Care and Use Committee on 02Feb2017.

## Figures and Tables

**Table 1:** Cell lines (31 in total) of this study grouped around several themes (invitromes).

Cell Line Designation	Grouping Cell Lines Based on the Properties of Either I or II					
	I. Sample from Which the Cell Line Was Developed				II. Cell Line Once Developed	
	I.A. Species Properties			I.B. Anatomy	II.A. General Properties	
	I.A1. Common Name	I.A2. Habitat	I.A3. Natural Geography	I.B1. Organ	II.A1. Cell Shape	II.A2. Availability
ACBA	Arctic Char	Anadromous	Arctic	Bulbous Arteriosus	Fibroblast	Informal ^1^
ASHe ^3^	Atlantic Salmon	Anadromous	North Atlantic	Heart	Endothelial	Informal
ASCF ^3^	Atlantic Salmon	Anadromous	North Atlantic	Caudal Fin	Fibroblast	Informal
ASimf20	Atlantic Salmon	Anadromous	North Atlantic	Gut	Myofibroblast	Informal
ASP309	Atlantic Salmon	Anadromous	North Atlantic	Pituitary	Epithelial	Informal
BAASf ^3^	Atlantic Salmon	Anadromous	North Atlantic	Bulbous Arteriosus	Fibroblast	Informal
HK13 *^,3^	Atlantic Salmon	Anadromous	North Atlantic	Kidney	Epithelial	Informal
CHSE-214 **	Chinook Salmon	Anadromous	Pacific Northwest	Embryo	Epithelial	Curated ^2^
RTG-2 **	Rainbow Trout	Anadromous	Pacific Northwest	Gonad	Fibroblast	Curated
RTgill-W1	Rainbow Trout	Anadromous	Pacific Northwest	Gill	Epithelial	Curated
RTH-149	Rainbow Trout	Anadromous	Pacific Northwest	Hepatoma	Epithelial	Curated
RTL-W1	Rainbow Trout	Anadromous	Pacific Northwest	Liver	Epithelial	Curated
RTgutGC	Rainbow Trout	Anadromous	Pacific Northwest	Intestine	Epithelial	Informal
RTHDF	Rainbow Trout	Anadromous	Pacific Northwest	Skin	Fibroblast	Informal
RTS11	Rainbow Trout	Anadromous	Pacific Northwest	Spleen	Macrophage	Informal
STB5 Gill ^3^	Threespined Stickleback	Anadromous	Pacific Northwest	Gill	Epithelial	Informal
STB6 Gill ^3^	Threespined Stickleback	Anadromous	Pacific Northwest	Gill	Epithelial	Informal
STBH ^3^	Threespined Stickleback	Anadromous	Pacific Northwest	Heart	Epithelial	Informal
PHL	Pacific Herring	Marine	North Pacific	Larvae	Epithelial	Curated
EPC **	Fathead Minnow	Freshwater	Great Lakes Basin	Skin	Epithelial	Curated
GarL	Gar	Freshwater	Great Lakes Basin	Liver	Epithelial	Informal
SB3	Lake Sturgeon	Freshwater	Great Lakes Basin	Brain	Epithelial	Informal
WE-Cfin11f	Walleye	Freshwater	Great Lakes Basin	Caudal Fin	Fibroblast	Informal
WE-Skin11f	Walleye	Freshwater	Great Lakes Basin	Skin	Fibroblast	Informal
WEBA	Walleye	Freshwater	Great Lakes Basin	Bulbous Arteriosus	Endothelial	Informal
YFP5	Yellow Perch	Freshwater	Great Lakes Basin	Fin	Fibroblasts	Informal
EelB	American Eel	Catadromous	North Atlantic	Brain	Endothelial	Informal
PBLE	American Eel	Catadromous	North Atlantic	Blood	Epithelial	Informal
HEW	Haddock	Marine	North Atlantic	Embryo	Epithelial	Informal
ZEB2J	Zebrafish	Freshwater	South Asia	Embryo	Epithelial	Informal
ZSSJ	Zebrafish	Freshwater	South Asia	Spleen	Epithelial	Informal

* HK13 was derived from a PRV-1 infected fish. Apart from originating from an infected fish, this cell line was not used in the in vitro screening of PRV-1 containing head kidney and red blood cell homogenates. ** Cell lines also evaluated by others for PRV-1 susceptibility. ^1^ Cell lines informally shared among researchers. ^2^ Cell lines are in curated culture collections. ^3^ Newly developed cell lines from this work.

**Table 2:** PRV-1 RNA levels (Ct values) in 11 cell lines exposed to Chilean PRV-1 (unknown subtype) head kidney homogenates.

		PRV-1 Ct * in Cultures at Four Time Points (Days) after Exposure			
Species	Cells Lines	7	14	21	28
Atlantic salmon	ASP309	32.9	Negative	Negative	Negative
	ASCF	32.5	Negative	Negative	Negative
	ASimf20	32.6	Negative	Negative	33.4
Fathead minnow	EPC	32.1	Negative	Negative	Negative
Haddock	HEW	33.7	Negative	Negative	Negative
Rainbow trout	RTgutGC	27.3	29.1	35.0	35.0
	RTG-2	35.0	35.0	34.2	31.1 **
	RTL-W1	Negative	Negative	Negative	Negative
	RTHDF	Negative	26.4 **	Negative	Negative
Walleye	WE-skin11f	29.5	Negative	Negative	Negative
	WEBA	30.6	Negative	34.6	Negative

* Ct = cycle threshold; Samples with Ct value ≥ than 35.0 or without Ct values are scored negative. Ct values are means of three biological replicates. ** Two out of three replicates were negative.

**Table 3:** PRV-1 RNA levels (Ct values) in 26 cell lines exposed to Canadian (PRV-1a) and Norwegian (PRV-1b) red blood cells homogenate in single and multiple passages virus isolation assays.

Species	Cell Line	Virus Isolation Assays	Passage Number of Collected Samples (Initial–Final)	Cumulative Days Post-Inoculation (Initial–Final)	Ct Value (Initial–Final)	
					Canada PRV	Norway PRV
American Eel	EelB	Single cell culture passage	1	2–14	30.7–30.9	32.6–32.7
		Sub-cultivating only exposed cells	1–2	22–43	27.6–28.9	27.2–30.1
	PBLE	Single cell culture passage	1	2–14	30.5–29.4 *	33.7–29.2 *
		Sub-cultivating only exposed cells	1–2	22–43	27.4–27.2	26.9–29.4
Arctic Charr	ACBA	Supernatant transfer	1–2	14–28	25.2–34.2	27.6 *–N
		Sub-cultivating only exposed cells	1–2	22–43	28.2–29.1	26.4–30.4
Atlantic Salmon	ASCF	Single cell culture passage	1	2–14	29.9–29.4	31.9–32.4
		Sub-cultivating only exposed cells	1–2	15–44	25.7–24.8	27.8–31.5
	ASHe	Single cell culture passage	1	2–14	30.8–30.9	33.5–32.1
		Sub-cultivating with fresh cells	1–2	14–28	31.4–31.3	No data
		Supernatant transfer	1–2	26–40	31.4–N	35.5 *–37.4 *
		Cell lysate transfer	1–2	14–28	22.2–25.4 **	25.7–27.1
		Sub-cultivating only exposed cells	1–2	15–44	28.8–31.0	28.8–32.8
	ASimf20	Single cell culture passage	1	2–14	28.4–28.4	32.1–32.2
		Supernatant transfer	1–2	26–40	25.0–37.1 *	27.2–N
		Cell lysate transfer	1–2	14–28	20.9–25.3	25.5–27.1
		Sub-cultivating only exposed cells	1–2	15–43	25.8–27.5	25.2–31.2
	ASP309	Supernatant transfer	1–2	26–40	24.4–31.2	28.2–34.9
	BAASf	Single cell culture passage	1	2–14	28.7–28.7	31.6–31.6
		Sub-cultivating with fresh cells	1–2	14–28	27.0–30.4	No data
		Cell lysate transfer	1–2	14–28	22.1–26.7	25.1–27.6 *
		Sub-cultivating only exposed cells	1–2	15–44	27.6–29.8	27.2–31.4
Chinook Salmon	CHSE-214	Supernatant transfer	1–2	14–28	27.3–33.2	30.5–N
		Cell lysate transfer	1–2	14–28	24.0–27.0 *	24.9–N
Fathead Minnow	EPC	Supernatant transfer	1–2	14–28	24.9–33.9 *	29.2–33.9
		Cell lysate transfer	1–2	14–28	25.5–28.2	27.1–31.9
Gar	GarL	Single cell culture passage	1	2–14	29.7 *–27.6	33.2 *–30.2
Pacific herring	PHL	Sub-cultivating with fresh cells	1–2	14–28	26.2–30.2	No data
Rainbow Trout	RTgill-W1	Supernatant transfer	1–2	14–28	26.9–32.4	29.8–33.8
		Cell lysate transfer	1–2	14–28	25.5–26.5	27.5–30.0
		Sub-cultivating only exposed cells	1–2	14–44	26.7–28.1	N–N
	RTgutGC	Single cell culture passage	1	2–14	30.8–28.3	31.0–32.0
		Sub-cultivating with fresh cells	1–2	14–28	26.0–N	No data
		Cell lysate transfer	1–2	14–28	24.6–28.1	26.9 *–30.2
		Sub-cultivating only exposed cells	1–2	14–44	27.4–29.2	28.5–33.2
	RTHDF	Single cell culture passage	1	2–14	31.7–30.2	34.0–34.9 *
		Sub-cultivating with fresh cells	1–2	14–28	31.6–31.3	No data
		Supernatant transfer	1–2	14–28	26.3 *–33.8	29.2–33.1
		Cell lysate transfer	1–2	14–28	22.9–26.6	26.5–28.7
		Sub-cultivating only exposed cells	1–2	14–44	28.1–29.4	27.0–31.6
	RTH-149	Single cell culture passage	1	2–14	29.5–26.6	33.0 *–N
		Sub-cultivating only exposed cells	1–2	15–43	24.7–24.7	28.9–32.4
	RTL-W1	Supernatant transfer	1–2	14–28	28.2 *–33.6	27.0–N
		Cell lysate transfer	1–2	14–28	24.9–27.0	26.9–N
		Sub-cultivating only exposed cells	1–2	14–44	28.0–28.5	28.7–33.5
	RTS11	Cell lysate transfer	1–2	14–28	22.6–27.0	26.9–28.8
		Sub-cultivating only exposed cells	1–2	15–43	22.6–23.8	25.8–26.0
Stickleback (Threespined)	STB5 Gill	Single cell culture passage	1	2–14	31.7–30.5	31.6–33.8
		Sub-cultivating with fresh cells	1–2	14–28	27.1–32.7	No data
	STB6 Gill	Sub-cultivating with fresh cells	1–2	14–28	23.8–28.3	No data
	STBH	Sub-cultivating with fresh cells	1–2	14–38	25.0–27.2	No data
Sturgeon	SB3	Supernatant transfer	1–2	14–28	24.4–32.7 *	28.7–N
Walleye	WE-cfin11f	Sub-cultivating only exposed cells	1–2	16–43	23.7–25.6	24.9–29.4
Yellow Perch	YPF5	Sub-cultivating only exposed cells	1–2	22–43	24.3–28.6	28.7–31.2
Zebrafish	ZEB2J	Single cell culture passage	1	2–14	27.7 *–27.6	31.0–32.2
		Sub-cultivating only exposed cells	1–2	16–43	26.1–28.4	27.1–30.8
	ZSSJ	Sub-cultivating with fresh cells	1–2	14–28	26.2–30.1	No data

Ct—Average of two biological replicates. N—Samples with Ct value equal to or greater than 35.0 and those without Ct values are considered as negative. * One of two biological replicates was negative. ** Only one biological replicate. No data—experiment not performed.

**Table 4:** PRV-1 RNA levels (Ct values) in seven cell lines co-administered with Canadian (PRV-1a) and Norwegian (PRV-1b) red blood cell homogenates.

	Cell Culture		Ct ^1^ after Exposures for	
Cell Line Designation	Species (Common Name)	Morphology	2 Days	14 Days
EelB	American eel	endothelial	27.7	28.5
PBLE	American eel	fibroblast	24.8	25.7
ASCF	Atlantic salmon	fibroblast	26.8	26.3
ASHe	Atlantic salmon	endothelial	27.7	29.4
ASimf20	Atlantic salmon	myofibroblast	27.5	27.7
BAASf	Atlantic salmon	fibroblast	27.6	27.3
RTgill-W1	Rainbow trout	epithelial	29.5	30.4

^1^ Ct = cycle threshold (Ct)—average of two biological replicates.

**Table 5:** PRV-1 RNA levels (Ct values) in eight cell lines co-administered with either Canadian (PRV-1a) or Norwegian (PRV-1b) red blood cell homogenates together with either CSV ^1^ or IPNV ^2^.

Cell Cultures			Ct ^3^ in Cultures at 2 Times after Initiation of Exposure	
Cell Line	RBC Homogenates	Co-Infected Virus	Passage 1 (13 to 18 Days ^4^)	Passage 2 [26 to 32 Cumulative Days ^5^ (12 to 14 Days after Passaging)]
EPC	Canada	CSV	22.4 *	32.0 *
	Norway	CSV	24.5	33.7
	Canada	IPNV	26.8	32.4
	Norway	IPNV	29.9	Negative
ASHe	Canada	IPNV	25.3	27.8
	Norway	IPNV	25.2	27.5
BAASf	Canada	IPNV	26.7	29.0
	Norway	IPNV	26.6	30.0
CHSE-214	Canada	CSV	21.5	30.4
	Norway	CSV	23.4	30.2
	Canada	IPNV	25.1	31.8
	Norway	IPNV	26.1	30.2
RTHDF	Canada	CSV	26.8	32.2
	Norway	CSV	29.3	Negative
	Canada	IPNV	27.9	32.6
	Norway	IPNV	28.0	32.8
RTgill-W1	Canada	CSV	24.7	28.8
	Norway	CSV	28.0	33.1
	Canada	IPNV	25.0	30.7
	Norway	IPNV	26.9	28.6
RTL-W1	Canada	CSV	24.1	31.6
	Norway	CSV	28.5	Negative
RTS11	Canada	CSV	Negative	32.8 **
	Norway	CSV	25.8 **	33.6 **
	Canada	IPNV	23.9	30.4 **
	Norway	IPNV	24.9	30.4 **

^1^ CSV =chum salmon reovirus at 10^4.3^ TCID_50_/mL. ^2^ IPNV= infectious pancreatic necrosis virus at 10^8.1^ TCID_50_/mL. ^3^ Ct =cycle threshold; samples with Ct value ≥ than 35 or without Ct values are scored negative. ^4^ For some replicate flasks after 13–18 days, RNA was extracted for RT-qPCR. For other replicate flasks after 13–18 days, the medium was collected & applied to fresh cultures and incubated further. ^5^ After 12–14 days of incubation with medium from passage 1, RNA was extracted for RT-qPCR. * All values are means of two biological replicates. ** One of two biological replicates was negative.

## Supplementary Materials

The following are available online at https://www.mdpi.com/2076-0817/9/10/833/s1, Table S1. PRV-1 RNA levels (Ct values) in Atlantic salmon infected with PRV-1a at PBS-DFO, Nanaimo, B.C. (trial 1) and PRV-1 (unknown subtype) at Elanco Canada, Victoria, P.E.I. (trial 2).
