# Supplementary material for: Screening of Fish Cell Lines for Piscine Orthoreovirus-1 (PRV-1) Amplification: Identification of the Non-Supportive PRV-1 Invitrome
Source: Pathogens. 2020 Oct 12;9(10):833. doi: 10.3390/pathogens9100833 (PMC7601784; doi:10.3390/pathogens9100833)
Supplement: Supplementary file 1 [file pathogens-09-00833-s001.pdf]

Table S1. PRV-1 RNA levels (Ct values) in Atlantic salmon infected with PRV-1a at PBS-DFO, Nanaimo, B.C. (trial 1) and PRV-1 (unknown subtype) at Elanco Canada, Victoria, P.E.I. (trial 2).

Atlantic salmon infection trial 1					
Week 2		Week 3		Week 4	
**Fish**	**Ct**	**Fish**	**Ct**	**Fish**	**Ct**
1	12.7	11	17.7	21	19.1
2	13.2	12	19.3	22	25.4
3	13.0	13	20.0	23	14.1
4	20.0	14	16.3	24	24.4
5	22.8	15	15.6	25	24.4
6	23.8	16	13.3	26	23.7
7	Pooled with #6	17	15.2	27	20.3
8	14.5	18	15.2	28	25.2
9	18.7	19	15.1	29	19.0
10	Pooled with #9	20	13.0	30	24.4

Atlantic salmon infection trial 2			
Week 2		Week 3	
**Fish**	**Ct**	**Fish**	**Ct**
1	33.0	10	19.2
2	32.0	11	24.0
3	31.6	12	28.2
4	35.8	13	23.8
5	31.9	14	25.0
6	31.5	15	21.4
7	23.8	16	24.3
8	32.2	17	23.6
9	29.7	18	32.5
